# Stroke risk factors and treatment variables in rural and urban Austria: An analysis of the Austrian Stroke Unit Registry

**DOI:** 10.1371/journal.pone.0214980

**Published:** 2019-04-10

**Authors:** Andrija Javor, Julia Ferrari, Alexandra Posekany, Susanne Asenbaum-Nan

**Affiliations:** 1 Department of Neurology, General Hospital Amstetten, Amstetten, Austria; 2 Department of Neurology, Hospital St. John´s of God, Vienna, Austria; 3 Department of Clinical Neuroscience, Danube University Krems, Krems an der Donau, Austria; 4 Gesundheit Österreich GmbH/BIQG, Vienna, Austria; University Lyon 1 Faculty of Dental Medicine, FRANCE

## Abstract

**Background and objectives:**

Differences in stroke risk factors and treatment variables between rural and urban regions in Austria were analyzed retrospectively as European data on this topic are scarce.

**Research design and methods:**

We performed statistical analysis using group comparisons and time series analysis of data of the Austrian Stroke Unit Registry between 2005 and 2016. 87411 patients were divided into three groups (rural, intermediate, urban) according to the degree of urbanisation classification of the European Commission/Eurostat.

**Results:**

Patients in the rural group were significantly younger, more often female, had a lower pre-stroke disability, and were more frequently transported by an emergency physician. Vascular risk factors were significantly higher in urban patients, leading to a higher rate of microangiopathic etiology. Onset-to-door (ODT) and Onset-to-treatment times were significantly higher in the rural group, but ODTs decreased over time. Door-to-needle times and time to first vascular imaging were significantly lower in the rural group. Intravenous thrombolysis and rehabilitation rates were lower in urban patients.

**Discussion and implications:**

Contrary to previous literature predominantly from outside of Europe, vascular risk factors were higher in Austrian urban patients. Further, rural patients had higher intravenous thrombolysis and rehabilitation rates maybe because of lower pre-stroke disability. ODTs in rural patients were generally higher, but they decreased over time, which might be a consequence of better education of the public in noticing early stroke signs, better transportation and education of emergency medical personnel, better advance notification to the receiving hospital and implementation of Stroke Units in rural areas.

## Introduction

Ischemic stroke is responsible for a significant portion of disease burden and deaths, but outcome and incidence rates vary significantly between countries, as well as urban and rural regions [1]. Disparities between urban and rural regions in stroke care are increasing, which makes this topic increasingly important from a public health perspective [2].

There is a lack of sufficient data on stroke in rural areas, especially in Europe. Moreover, European evidence is largely of small scale [3–6]. A possible reason might lie in the fact that epidemiological data from Europe is confounded by a selection bias as most studies are published on data collected from only a few countries and mostly of urban populations [7]. Data from other world regions hint to suboptimal care in rural regions [8]. This might be explained by the level of education in recognition of stroke symptoms by the population, paramedics training, and transit times to hospitals [9] or the fact that patients in rural regions were less likely treated in Stroke units and to receive quick brain and vascular imaging, as well as consultations from neurologists and therapists and rehabilitation [10,11]. In addition, there seem to be differences in stroke risk factors between rural and urban populations [12].

Given the lack of data about differences in risk factors, management, and outcome of stroke in European rural and urban regions, we analyzed the respective data from the Austrian stroke registry.

## Methods

The Austrian Stroke Unit Registry prospectively collects data on standard characteristics, management, and outcome of stroke patients admitted to one of the currently 38 Austrian Stroke units. It is financed by the Federal Ministry of Health and is centrally administered by the Gesundheit Österreich GmbH. Stroke-relevant data is documented since 2003 in an anonymized fashion and scientific analyses have to be approved and supervised by an expert committee. Data entry, data protection, administration, and scientific analysis are regulated by the Federal Law on Quality in Health, the Federal Law on Gesundheit Österreich GmbH § 15a, and the Stroke Unit Registry Act.

**Fig 1:**
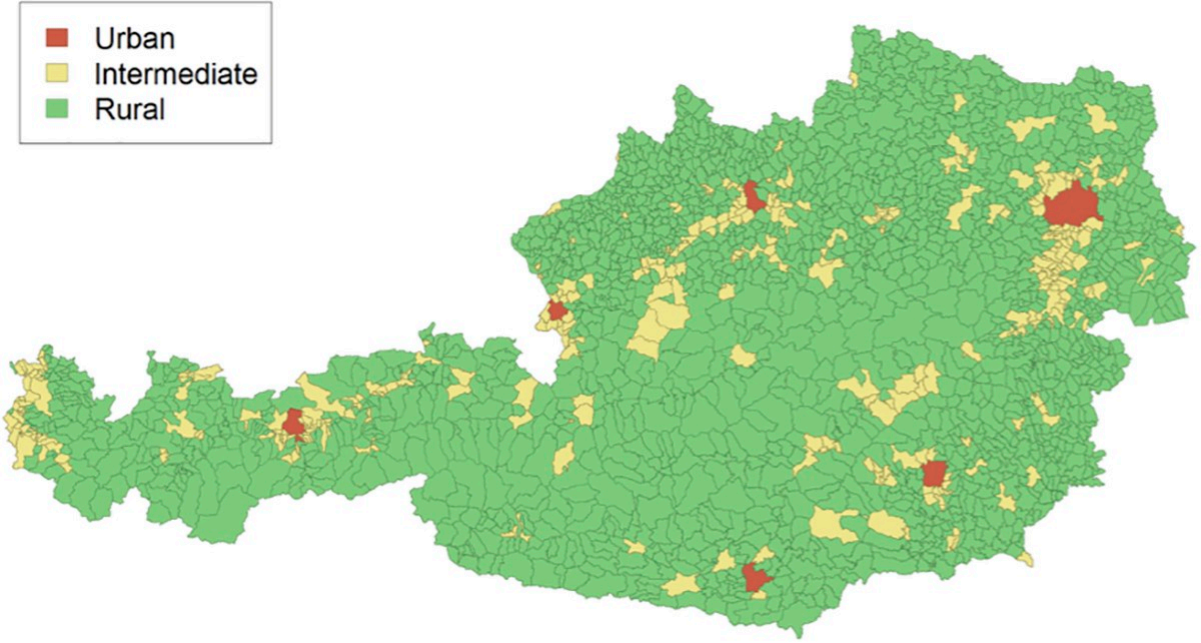
Visualization of urban, intermediate and rural areas in Austria.

This study analyzed registry data from 2005 to 2016 due to the low number of established Stroke units in Austria between 2003 and 2005. At the time of analysis there were n = 144 419 data sets available in the registry, from which n = 103 810 corresponded to ischemic strokes. Excluding those where no geographic information was available, n = 87 411 cases were finally included. Using the postal code of each patient we categorized all data sets into 3 groups according to the degree of urbanisation (DEGURBA) classification of the European Commission/Eurostat. This classification is based on the population size and density and contiguity of local administrative units level 2/municipalities (LAU2)–for a medical study using the same classification, see[13]. In a second step, these LAU2 are classified in the following sense: densely populated area (alternate name: cities or large urban area) with at least 50% of the population living in high-density clusters, intermediate density area (alternate name: towns and suburbs or small urban area) with less than 50% of the population living in rural grid cells and less than 50% lives in a high-density cluster, and thinly populated area (alternate name: rural area) with more than 50% of the population lives in rural grid cells (Fig 1). Applying this methodology on our data, the 3 groups consisted of the following numbers of data sets: urban n = 28 640, intermediate n = 21 505, and rural n = 37 266. Of these cases, n = 11 057, n = 7 118, and n = 11 122 in the 3 respective groups (urban, intermediate, rural) were reached for a follow-up telephone interview 3 months after the incident. The interviews were performed with either the patient and/or the care giver, or the treating physician.

The following variables of the Austrian Stroke Registry were included in the analysis: age, gender, risk factors (arterial hypertension, diabetes, previous stroke, heart attack, hypercholesterolemia, atrial fibrillation, other cardiac diseases, peripheral artery occlusive disease, smoking, regular alcohol consumption, acute alcohol intoxication), several other pre-hospital variables, such as the modified Rankin scale (mRS) before the event, mode of transportation (ambulance with/without emergency physician or other), as well as the onset-to-door time (ODT); variables at and during admission to the Stroke unit, such as National Institutes of Health Stroke Scale (NIH SS) and mRS, etiology (e.g. microangiopathic, macroangiopathic, cardioembolic, others, unkown), door-to-needle time (DNT), onset-to-treatment time (OTT), the time to first cerebral and vascular imaging, the intravenous (iv) thrombolysis rate, and the rate of interventional endovascular treatment. Finally, outcome variables, NIH and mRS at discharge from the Stroke unit, mortality rates, and referral rates to rehabilitation, were analyzed. Analyzed data from the follow-up interview include mRS and rehabilitation rates. For all time points patients with mRS 0 and 1 were summed as well as patients with mRS 2–5.

The statistical analyses were performed with the software package R. Comparisons between groups included χ^2^-tests and Kruskal-Wallis tests. We corrected for multiple comparisons using the Bonferroni-method. The level of significance was at least p < .001. Furthermore, the time series of ODTs were modeled by an autoregressive model. We applied model selection with AIC for determining the appropriate order of the autoregressive time-series models. Finally we assessed the clinical relevance of our statistically significant findings by looking at the relative difference of parameters between the urban and the rural group.

## Results

A detailed overview of all results is delineated in Tables 1–3. In summary, there was a significant difference in age and gender (each p<0.001), with higher age in the urban compared to the other groups [median age urban (Q_0.25_, Q_0.75_): 74.3 years (63.5, 83.2), intermediate: 73.4 (63.1, 81.5), and rural group: 73.7 (63.1, 81.3)] and a higher rate of females [urban: 14715 (51.4%), intermediate: 9999 (46.5%), rural group: 17123 (45.9%)].

**Table 1:** Analysis of risk factors for stroke.

Variable	Urban group	Intermediate group	Rural group	Relative difference ^+^
*(N*, *%)*				*%*
Arterial hypertension*	21058 (80.5)	15405 (78.4)	26904 (78.7)	2.29
Diabetes*	7158 (27.4)	4778 (24.3)	7956 (23.3)	17.60
Prior strokes*	6780 (25.9)	4344 (22.1)	7368 (21.6)	19.91
Prior heart attacks*	2886 (11)	1751 (8.9)	2692 (7.9)	39.24
Hypercholesterolemia*	15290 (58.4)	10325 (52.5)	18098 (52.9)	10.40
Atrial fibrillation	6969 (26.6)	5105 (26)	9222 (27)	-
Other cardiac disease	5851 (22.4)	4167 (21.2)	7646 (22.4)	-
Peripheral artery disease*	2132 (8.1)	1281 (6.5)	2149 (6.3)	28.57
Smoking*	6200 (23.7)	3248 (16.5)	4869 (14.2)	66.90
Regular alcohol consumption*	2395 (9.2)	1398 (7.1)	2374 (6.9)	33.33
Acute alcohol intoxication	131 (0.5)	104 (0.5)	162 (0.5)	-

^+^ Relative difference: between urban and rural group in %

* Level of significance at least p<0.001

**Table 2:** Etiology, treatment and time variables.

Variable	Urban group	Intermediate group	Rural group	Relative difference ^+^
*(N*, *%)*				*%*
Microangiopathy*	7031 (24.5)	4471 (20.8)	7935 (21.3)	15.02
Macroangiopathy	2577 (9)	2642 (12.3)	4190 (11.2)	-
Cardioembolic	6070 (21.2)	4747 (22.1)	8771 (23.5)	-
Others	629 (2.2)	402 (1.9)	670 (1.8)	-
Unkown	12333 (43.1)	9243 (43)	15700 (42.1)	-
Thombolysis rate, iv* (%)	3733 (14.3)	3403 (17.3)	5314 (15.6)	9.09
Endovascular treatment (%)	362 (4.6)	217 (4)	366 (3.7)	-
Referral rate to rehabilitation*	3725 (38.1)	2444 (39.9)	3856 (41.1)	7.87
*Time variables (median in min*, *Q*_*0*.*25*_, *Q*_*0*.*75)*_				
ODT*	105 (60, 253)	118 (60, 260)	120 (65, 261.2)	14.29
DNT*	48 (32, 70)	48 (30,71)	45 (30, 70)	6.67
OTT*	120 (92, 165.5)	123 (90, 170)	130 (100, 173)	8.33
Time to first cerebral imaging*	30 (17, 61)	30 (15, 69)	30 (15, 60)	0
Time to first vascular imaging*	240 (60, 1263)	100 (40, 900)	105 (40, 940.5)	128.57

^+^ Relative difference: between urban and rural group in %

* Level of significance at least p<0.001

iv = intravenous, ODT = onset-to-door time, DNT = door-to-needle-time, OTT = onset-to-treatment time

**Table 3:** Summary of pre- and post-Stroke disability variables.

Variable	Urban group	Intermediate group	Rural group	Relative difference ^+^
*Before event*				*%*
mRS before event < = 1* (N, %)	21572 (75.6)	17231 (80.5)	29797 (80.7)	6.75
mRS before event > = 2 (N, %)	6967 (24.4)	4183 (19.5)	7117 (19.3)	-
*Admission*				
NIH SS* (median, Q_0.25_, Q_0.75_)	4 (1, 8)	4 (1, 8)	4 (1, 8)	0
mRS < = 1 (N,%)	8055 (28.2)	6156 (28.7)	10570 (28.6)	-
mRS> = 2 (N,%)	20484 (71.8)	15258 (71.3)	26344 (71.4)	-
*Discharge*				
NIH SS*(median, Q_0.25_, Q_0.75_)	2 (0, 5)	2 (0, 5)	2 (0, 5)	0
mRS < = 1 (N,%)	11258 (43.6)	8692 (44.7)	14631 (43.6)	-
mRS> = 2 (N,%)	14568 (56.4)	10771 (55.3)	18918 (56.4)	-
Mortality (N,%)	707 (2.7)	430 (2.2)	803 (2.4)	-
*Follow-up at 3-months*				
mRS < = 1* (N,%)	5427 (48.5)	3595 (52.2)	5519 (51.8)	6.80
mRS> = 2 (N,%)	5766 (51.5)	3288 (47.8)	5128 (48.2)	-

^+^ Relative difference: between urban and rural group in %

* Level of significance at least p<0.001

mRS = modified Rankin scale, NIH SS = National Institutes of Health Stroke Scale

Further, a significant difference in all risk factors between patients groups was found (p<0.001), except atrial fibrillation, other cardiac diseases and acute alcohol intoxication (Table 1). Etiology of stroke differed significantly between groups, with a higher rate of microangiopathic strokes in urban regions (p<0.001) (Table 2).

**Fig 2:**
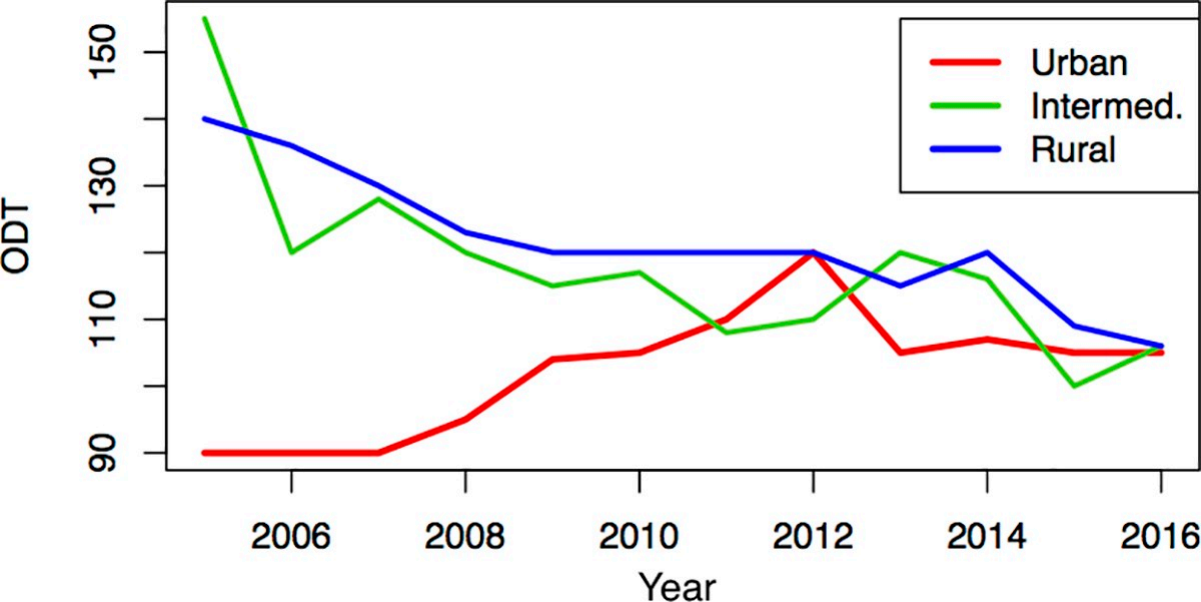
Development of Onset-to-door (ODT) times during the observation period of this study.

A significantly higher portion of patients in the urban group was transported to hospital by an ambulance without supervision by an emergency physician than in intermediate or rural areas [urban n = 18080 (63.2%); intermediate n = 8537 (39.7%); rural n = 14588 (39.2%)]. ODT and OTT were significantly higher in the rural group (p<0.001), whereas DNT was significantly higher in the urban group (p<0.001) compared to the respective other groups. Times to first cerebral (p = 0.009) and vascular imaging (p<0.001) were significantly higher in the urban group (p<0.001) (Table 2). Autoregressive time series models revealed that for ODTs of the rural and urban group a first order autoregressive model is preferred, while a second order model was preferred for the intermediate group. ODTs of the intermediate and rural group showed a decreasing trend over time, while the urban group varied around a time-independent mean without any trend (Fig 2).

Treatment variables differed significantly between groups, with a higher iv thrombolysis and referral rate to rehabilitation in the rural group (both p<0.001), whereas the rate of endovascular treatment did not differ significantly (p = 1.000).

An analysis of severity of stroke and disability variables before, during and after Stroke unit treatment (Table 3) revealed a lower disability of rural patients (measured through the mRS) before the event and 3-months after the event (both p<0.001), but not at admission or discharge from the Stroke unit (both p = 1.000). Median NIH SS reached 4 points in all groups at admission and 2 in all groups at discharge.

Relative differences between rural and urban group were listed in Tables 1–3.

## Discussion

In this study we analyzed data from the Austrian stroke Registry in order to contribute data from a high-income European country on differences between rural and urban populations in stroke risk factors and treatment variables, because until now literature was dominated by studies on non-European and low-income countries, and these findings cannot be easily translated to high-income countries [14].

Our analysis revealed that urban patients in Austria show a different risk profile compared to those living in intermediate or rural areas. In detail, they had higher rates of arterial hypertension, diabetes, hypercholesterolemia, smoking, alcohol consumption, peripheral artery occlusive disease, prior heart attacks and strokes. All these variables showed a relative difference between groups about 20% and more percent, except for arterial hypertension where the relative difference was small. Congruently, pre-stroke disability and microangiopathic stroke etiology was lower in rural patients compared to the other groups, although the relative difference in pre-stroke disability was rather small. Interestingly, this is contrary to previous data revealing higher rates of hyperlipidemia and prior stroke in rural patients in China [12] and a higher Body-Mass-Index, a more sedentary lifestyle, and higher cholesterol levels in a Swedish sample of rural inhabitants [15]. We think that there are several possible explanations for this: First, health systems and several socioeconomic variables, such as income, education etc. differ between Austria and developing countries, especially in rural populations, which might lead to a healthier lifestyle and higher focus in preventive medical measures in rural Austria. Second, the Swedish sample was of smaller size and not focusing on stroke patients, which might explain differing results. Finally, we observed differences in some variables, such as age, gender, and NIH scores at admission and discharge between groups, but they were low in absolute values and relative differences implying low clinical significance. Nevertheless, the overall consistent pattern between risk factors and etiology in the groups makes us confident that these differences are indeed clinically relevant.

Another interesting aspect of our analyses was ODTs differing significantly between the groups at the beginning, but not at the end of the observation period due to a decreasing trend. Overall a high relative difference of ODT was obvious. One could hypothesize that this trend follows the evolution in patient transport due to the technical development and advances in helicopter availability in recent years [16]. Furthermore, decreasing ODTs might be a consequence of better education of the general public in noticing early stroke signs, education of emergency medical service personnel, and advance notification to the receiving hospital [17] or the implementation of Stroke units in rural Austrian regions in recent years [18]. An inverse relation was found for DNT and times to first cerebral and vascular imaging in our study with higher times in urban than rural patients. While the relative difference for DNT and the time to cerebral imaging was small, it was implying high clinical significance for the time of first vascular imaging. This might be explained by differences in hospital sizes with shorter within hospital distances between the emergency room, imaging facilities and the Stroke unit in rural areas.

Furthermore, we found a statistically and clinically significant difference in iv thrombolysis rates with more rural patients receiving this treatment. In a Canadian sample, no such difference could be found [11] and actually most evidence hints to poorer health care in rural hospitals [19–22]. Higher iv thrombolysis rates and higher OTTs at once in the group of rural inhabitants are indeed an interesting result that cannot be easily explained. In fact, differing stroke severity cannot have influenced these results as our analyses of disability ratings at admission and discharge from the Stroke unit show a similar degree of stroke severity. A possible explanation might be the fact that rural inhabitants had a lower pre-stroke disability leading to a lower rate of contraindications for iv thrombolysis. Lower pre-stroke disability could, in turn, be a consequence of lifestyle differences and subsequent lower cardiovascular risk factors and prior heart attacks/strokes in rural patients. On the contrary, the rate of endovascular treatment was not significantly different between groups. Although this analysis is based on a far lower number of data points, it is nevertheless in line with recent evidence from Austria [23].

The limitations of our study evolve out of the data analyzed: First, the Austrian Stroke Unit Registry only includes patients that are treated at Stroke units and not those being admitted to hospitals without a Stroke unit, to the general ward due to clinical characteristics or capacity reasons, or do not consult a doctor at all. On the one hand, one might speculate that such cases are more prevalent in rural than in urban regions and therefore data of the registry is biased. On the other hand, the number of overall stroke patients treated in Stroke units in Austria increases constantly and was over 60% in 2013 [24]. However, the relevant Austrian treatment recommendations emphasize Stroke unit treatment as being the standard of care and limits iv thrombolysis to the Stroke unit setting. We believe that risk factors for strokes treated outside of stroke units should not be significantly different of our stroke unit patient sample. Finally, treatment decision-making (e.g. inclusion and exclusion criteria for iv thrombolysis) should not differ significantly between urban and rural regions of Austria. In light of these considerations, we think that the Austrian Stroke Registry data are highly suited to be the basis of our analyses[25]. Second, the data available in the registry are regulated by law and therefore other possibly relevant variables not covered by the registry, such as ethnicity, socioeconomic status, or physical activity, were not available for analysis and could not be accessed due to anonymity. Third, our study is not population based in an epidemiological sense. Forth, the division of data into groups was based upon the postal code of patients. Even though this methodology has been applied before [13], it cannot be excluded that some patients might have been treated in a hospital in another area than their hometown/city, but this should apply to all groups to a similar extent. Further, each Stroke unit in Austria has a determined catchment area, which makes it most probably that patients are treated in the Stroke unit nearest to the postal code of residence.

We think that our results contribute significantly to existing literature on differences in stroke risk factors and treatment variables between rural and urban regions as prior data on European countries, especially of high-income, was limited. European countries have different geographical characteristics compared to other high-income countries in North America and Australia and huge differences in health systems exist when compared to low-income Asian or African countries. Therefore implications for Europe cannot be drawn out of data collected on those continents. Contrarily, we believe the results of our study based on Austrian data could be applicable to other countries with a similar geography, health care system, socioeconomic status of inhabitants etc, such as Germany, Switzerland, and other Western European countries.

Our results favor the implementation of preventional measures concerning cardiovascular risk factors in order to promote public health concerning stroke especially in urban regions of Europe. Even though investments in the development of transportation have already led to significant improvements in stroke management of rural patients in the past, a further reduction of ODTs in order to reach the urban ‘benchmark’ is necessary. Moreover, we call for further studies in other European countries in order to demonstrate comparability of our results. Finally, it will be interesting to see how recent developments in technique and accessibility of endovascular treatment will influence treatment and outcome variables of urban and rural regions in Austria.
